# Sliding Wear Performance of Natural Fiber–Reinforced Polymer Matrix Composites

**DOI:** 10.1155/tswj/6617516

**Published:** 2026-03-20

**Authors:** Rajdeep Paul, Sumit Das Lala, Payel Deb, Abhijit Bhowmik, Nagaraj Ashok

**Affiliations:** ^1^ Department of Mechanical Engineering, National Institute of Technology, Agartala, Tripura, India, int.gov.br; ^2^ Department of Mechanical Engineering, PIET, Parul University, Vadodara, Gujarat, 391760, India, paruluniversity.ac.in; ^3^ Department of Additive Manufacturing, Mechanical Engineering, SIMATS, Saveetha Institute of Medical and Technical Sciences, Thandalam, Chennai, 602105, India, saveetha.com; ^4^ Centre for Research Impact and Outcome, Chitkara University, Rajpura, 140401, Punjab, India, chitkara.edu.in; ^5^ Faculty of Mechanical Engineering, Jimma Institute of Technology, Jimma University, Jimma, Ethiopia, ju.edu.et

**Keywords:** friction, lubrication, natural fiber, polymer resin, tribology, wear

## Abstract

While synthetic fiber composites offer some positive environmental attributes, researchers are trying to explore natural fiber composites (NFCs) due to the high cost and pollution associated with their production. As a result, it is essential to look at the tribological properties that natural composite materials exhibit. This research aims to provide a thorough examination of the current literature about the tribological characteristics of particle‐reinforced and fiber‐reinforced natural composites under lubrication, such as volume loss, friction, and wear. Additionally, the operational and material aspects influencing tribological behavior are also examined in this study. The results show that a wide range of material parameters, including particle size, volume fraction, fiber orientation, fiber length, surface treatment, and aspect ratio, as well as numerous operational factors, including normal load, sliding velocity, sliding distance, and temperature, significantly affect the tribological properties. The current review study, which focuses on the tribological characteristics of NFCs in lubricated environments, is assumed by the authors to have the ability to direct future research in the creation of innovative material designs for tribological applications.

## 1. Introduction

In recent years, various global organizations have been emphasizing the significance of technical industries such as energy, pollution, transportation, and agriculture in adopting green engineering approaches for the production of their goods [1]. Furthermore, previous industry surveys have provided compelling evidence that the marketing plan anticipates a significant increase in demand for eco‐friendly products in the near future. Presently, the automotive and aerospace sectors are directing their attention toward the development of novel material designs that possess decreased weight while maintaining their specialized strength [2].

Furthermore, these novel materials provide characteristics that enable enhanced durability and energy efficiency through augmented wear resistance and coefficient of friction (COF). Given these considerations, it can be observed that various sectors heavily rely on the advancement of fiber as well as filler‐reinforced polymer composites to attain optimal product performance while maintaining cost efficiency [3, 4]. Artificial fibers/fillers with enhanced tensile strength, such as glass, aramid, carbon, Kevlar fibers, wood dust, and sawdust, are frequently employed as reinforcing agents in diverse polymer matrices, encompassing thermoset, thermoplastic, and elastomeric materials [5–8]. These composite materials are widely utilized in many engineering applications that include supporting and bearing loads in structural systems. In addition to their utility in structural applications, these composites have the potential to be highly successful in bearing applications. Additionally, the extended lifespan of worn parts can contribute to cost‐effective operation through decreased maintenance requirements. Nevertheless, the ecological crisis resulting from nondegradable components and the greenhouse effect has led to significant drawbacks. Consequently, the issue of global warming has emerged as a prominent worry in the realm of novel material designs [9, 10]. Table 1 represents the chemical and mechanical properties of natural fibers.

**TABLE 1:** Chemical and mechanical properties of natural fibers.

Types of fiber	Chemical properties				Mechanical properties			
	Cellulose (wt%)	Hemicellulose (wt%)	Lignin (wt%)	Moisture content (wt%)	Tensile strength (MPa)	Young’s modulus (GPa)	Specific strength	Elongation (%)
Agave	68.4	15.5	4.9	7.7	5.7	—	4.7	—
Kenaf	44	20	20	—	223	15	186	2.5–3.5
Bamboo	48–74	12–70	10–20	1.4	1.8	12	2.6	—
Jute	40–50	13–20	12–24	1.5–1.8	400–780	27	365	1.2–1.5
Ramie	68–75	15	0.6	3.5–3.8	400–940	62–130	—	—
Coir	36–43	10–20	40–45	3–4	595	4–6	494	—
Softwood	40–45		35	4.5	1000	40	667	—
Flax	70–86	16	1–4	8–12	350	28	—	1.5–1.8
Hemp	70–75	18–22	4–6	6–12	390	35	—	1.8
Abaca	55–60	—	12–14	5–10				
Cotton	80–85	5–6	10–30	5–6	287–800	5–13		7–8
Betel nut	35–65	28–33	13–25	9–15	120–170	1.5–3		22–25
Oil Palm	42–65	17–33	13–25	12–25	50–400	0.6–2		4–18
Sugarcane	28–55	20–36	21–24	—	170–350	5–6		6.2–8
Banana	63–67	18–20	4–6	5–7	710–790	4–33		2.5–3.5
Palf	70–80	—	5–12	3–5	410–1600	35–82		1.6
Sawdust	40	20	30	5–10	16.22	2	—	—
Wood dust	41.58	32.81	33.56	3.08	28.29	1	—	—
Rice husk	25–35	18–21	26–31	5–10	29.83	3.2	—	—
Rubber seed shell	71.64	24.56	2.98	3–4	41	1.4	—	—
Walnut seed shell	30	24.9	35	5–10	36	1.2	—	—

Due to their unique advantages and exceptional performance, natural fiber–reinforced polymer composites (NFPCs) are increasingly being adopted in various applications. The industry frequently employs a variety of natural reinforcements, including standard fibers like jute, palm, and flax, as well as biomass fillers like sawdust, betel nut, and sugarcane residues. Improved mechanical properties, lower energy consumption, decreased density, and cost‐effectiveness are just a few benefits of using natural fibers and fillers [11]. Conversely, natural fibers and fillers have their limitations as well. These materials’ ability to draw in moisture makes it easier for a weak bond to form between the fiber and polymer resin. Chemical treatments must be applied to increase natural fibers and fillers [12]. The performance of NFPC is influenced by several factors, including structure, physical characteristics, microfibrillar angle, defects, chemical characteristics, cell dimension, and the interaction of the fiber with the matrix [13–17]. The boundary that the fiber–matrix interface provides, where stress is passed from the matrix to the fiber, has been identified by researchers to be essential to the mechanical efficiency of NFPC [18]. Studying natural fiber features, such as impurities [19], moisture absorption [20], orientation [21], volume fraction [22], and physical properties [23], can help determine the mechanical capabilities of nonfungible polymer (NFPC) to a considerable extent. Young’s modulus and flexural modulus of natural fibers are determined by cellulose, which is the main constituent. Significantly more important than hydroxyl groups in controlling water absorption is hemicellulose [24–26]. Several studies [27–29] looked at various composite surface treatments. The principal objective of surface treatments for natural fibers is to enhance the fiber/matrix interfacial contact and optimize the composite’s efficient stress transferability. Chemical treatments include alkali treatment and chromium sulfate solution treatment [30], silane [31], benzoylation [32], permanganate [33], acrylation, acetylation, grafting acrylonitrile and stearic acid [34], coupling agent [35], non‐ionic detergent and stearic acid [36], pyridine or dichloromethane solvents using oleoyl chloride as a coupling agent [37], isocyanate [38], fatty acid derivative [39], and triazine [40, 41]. The application of NFPC in several engineering areas is growing quickly. Different kinds of NFPC are valued highly by companies like German Automotive Company, Proton Company, Cambridge Automotive Industry, and so forth. Outside of the automobile industry, natural fiber composites (NFCs) have found extensive application in the aerospace and construction sectors, as well as in sports equipment such as window panels, decking, bicycle frames, and so on [42–44].

## 2. Tribology in Materials

Tribological phenomena, including friction and wear, have been identified as significant factors contributing to material failure and equipment damage [45]. The energy consumption attributed to tribological interactions is estimated to account for around 23% of worldwide energy consumption. Within this figure, 20% of the energy is expended in efforts to mitigate friction, while the remaining 3% is allocated toward the replacement and reproduction of damaged equipment and spare parts resulting from wear [46]. The economic and environmental impact of tribological events associated with the mining industry was also examined by Holmberg et al. [47]. A study revealed that the friction and wear encountered throughout the mineral mining process result in an annual financial loss of 210,000 million euros, with 40% of this amount being allocated toward mitigating the effects of friction. In contrast, it has been observed that the global mining industry contributes to an annual release of around 970 million tons of carbon dioxide (CO2) as a result of friction and wear. This amount accounts for approximately 2.7% of the total global emissions [47]. Through the study and analysis of tribology, it is possible to greatly mitigate the losses resulting from the wear and friction that occur when two solid bodies are in motion relative to each other. Tribological investigations offer significant benefits in terms of enhanced efficiency, productivity, dependability, safety, and reduced occurrences of technical system failures [48, 49]. The effectiveness and dependability of mechanical systems depend heavily on the choice of appropriate contact materials and lubricants, if any. Contact between two bodies is typically where a tribologist’s attention is directed [50]. Friction and wear cannot occur without actual contact [51]. From a geometric standpoint, two fundamental forms of macro contact may be identified due to the relative motion of two contact surfaces: conformal and nonconformal [52]; the difference between the two lies in the stress–strain extent [53, 54]. Friction has been researched alongside the phenomenon of contact throughout history. It is well‐known that friction results in the loss of energy. Microfractures and surface wear can occur due to friction in microcontacts [55]. The SEM images as depicted here show the lower deformation and distortion for neat epoxy samples. However, at 2.5% wt of filler, the wear in composites is further reduced. This is because neat epoxy acts as a brittle material, whereas filler‐incorporated composite material shows better wear properties. With a further increase in filler content of around 12.5% wt, a high agglomeration is observed that also causes a lot of wear and tear in composites, as observed from the figure below.

**FIGURE 1:**
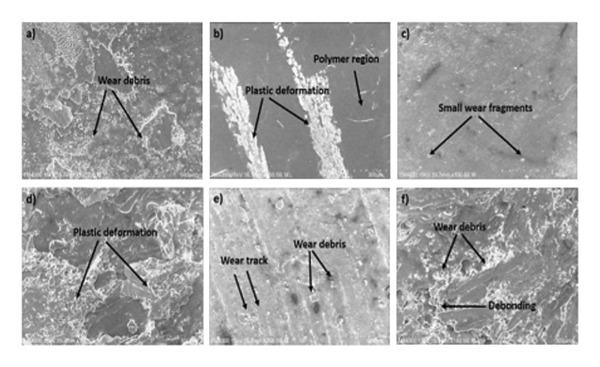
Surface morphology of the treated jute–epoxy composites observed under a microscope. The images depict (a) neat epoxy at 5 N, 3000 m; (b) neat epoxy at 15 N, 3000 m; (c) 2.5% FP composite at 5 N, 3000 m; (d) 2.5% FP composite at 15 N, 3000 m; (e) 12.5% FP composite at 5 N, 3000 m; and (f) 12.5% FP composite at 15 N, 3000 m [55].

The tribological properties of mechanical systems are significantly affected by different heat regimes and stress states, as evidenced by a substantial body of research [56–60]. It is widely recognized that frictional work during the wear process generates heat, leading to an inevitable rise in temperature at the contact interface. As demonstrated by Lim and Ashby [61], this thermal elevation is critical because it alters the mechanical properties of the sliding surfaces. Consequently, Amiri and Khonsari [62] noted that these thermally induced mechanical changes significantly dictate the tribological behavior of contact pairs. Tribology analysis in polymer composites is depicted in Figure 1.

**FIGURE 2:**
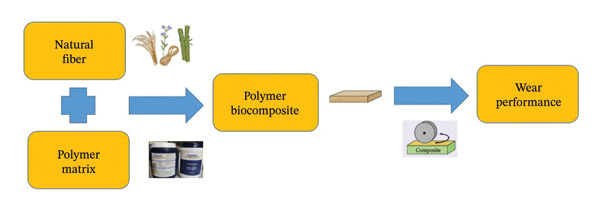
Tribology analysis in polymer composites.

In the specific context of polymer composites, friction and wear performance are driven by operational variables such as applied load (AL), sliding velocity (SV), and sliding distance (SD). Although the inclusion of natural fibers can sometimes lead to higher wear rates, the wear resistance of these materials can be substantially enhanced by using chemical treatments to improve the interfacial bonding between the fiber and the matrix [63]. The shearing zone’s wear behavior is also monitored using solid lubricants. Wear refers to the gradual deterioration of a surface due to the frictional forces exerted by the moving parts beneath it. Dimensional changes caused by wear lead to decreased efficiency and increased noise, vibration, and misalignment [64]. A wide variety of tribometers are employed by researchers for wear and friction studies, such as the pin‐on‐disc, block‐on‐disc, pin‐on‐drum, block‐on‐ring, linear tribometer, and dry sand rubber wheel apparatus [65–70]. When considering a composite material in a tribological contact, four specific fiber orientations can be defined based on their alignment relative to the sliding path: (a) parallel orientation (P‐O); (b) antiparallel orientation (AP‐O); (c) normal orientation (N‐O); and (d) random orientation (R‐O). Figure 2 shows the concept of tribological testing in polymer composites.

## 3. Types of Tribological Tests

**FIGURE 3:**
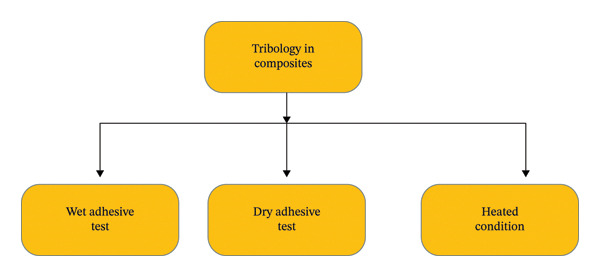
Tribology in polymer composites.

Generally, tribology tests can be conducted on dry, wet, and heated contact conditions. In dry conditions, the workpiece and counterface surface are directly in contact with each other, and there is no coolant or preheating done on the testing surface. In wet conditions, a continuous flow of lubricating medium such as water, oil, kerosene, engine oil, and so on is supplied to the testing surface. In the case of a heated condition, the counterface surface is preheated at a high temperature, and then the sample is placed upon the counterface material, and the wear of the testing sample is measured. Tribology in polymer composites is shown in Figure 3. In this review study, the tribological behavior of natural fiber/filler composites under both conditions has only been considered.

## 4. Discussion

### 4.1. Wet Adhesive Test

Adhesive wear occurs when bits of a soft surface break off and stick to another surface as two bodies slide past each other. Wet tests involved filling the container with the lubricating medium beforehand, which may be water, oil, kerosene, engine oil, vegetable oil, and so on. Each test is generally performed using clean lubricating oil to eliminate the possibility of contamination from previous experiments. To prevent oil from splashing out of the container and to maintain oil levels above the specimens, the containers are sealed during the testing. To overcome the challenge of indeterminate weight loss observed at low SV and distance, the experiments were performed at higher sliding speeds and distances than those used for the dry contact condition. Yousif et al. (2008) investigated the use of betel nut fiber as a reinforcement in polyester composites for tribological applications [71]. They found that the specific wear rate (SWR) varied significantly across the range of ALs and principal SDs tested. A slight increase in the SWR was noted as the SD increased. The research suggests that the decrease in material removal with increasing distance is likely due to the strong bonding between the fiber and the matrix. A significant decrease in the COF was reported during wet interactions compared to dry interactions. The presence of a liquid medium facilitates the reduction of contact between the asperities in the links during sliding. In the wet condition, the frictional and wear performance of the composite material reinforced with betel nut fibers exhibited a significant improvement of 94% and 50%, respectively. The scanning electron microscope (SEM) image revealed that debonding of fibers and fracture of fibers were detected under dry conditions. In the presence of moisture, fiber bundles exhibit no signs of fiber splitting or severe damage under conditions of high loading and increased SD. The friction coefficient versus SD under dry conditions is shown in Figure 4.

FIGURE 4Friction coefficient versus sliding distance under dry conditions for (a) betel nut composite and (b) CFRP composite [71, 72].

**Figure:**
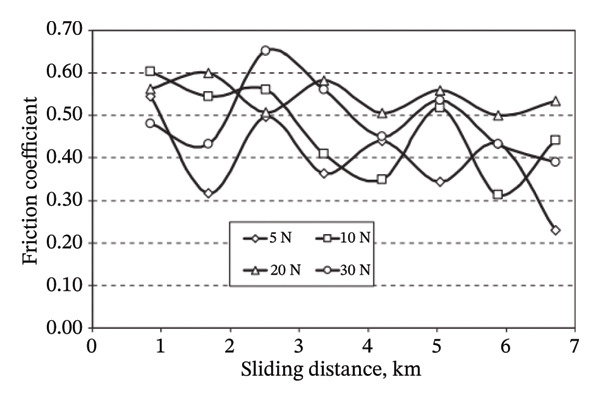
(a)

**Figure:**
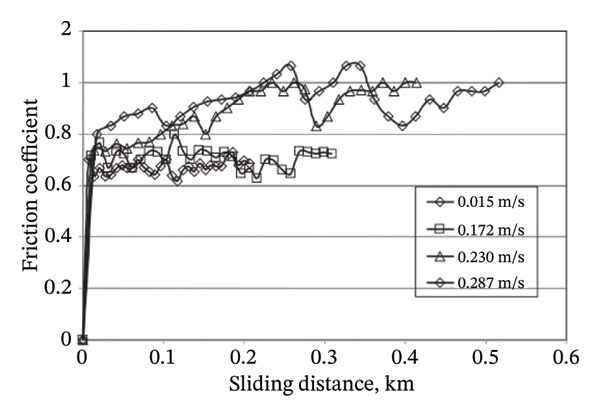
(b)

The tribological behavior of a coconut fiber–reinforced polyester (CFRP) composite was studied by Danaelan and Yousif in a dry/wet situation [72]. It was discovered that the COF is minimal at low speeds and grows with increasing velocity. COF values ranged from 0.70 to 0.90 in dry conditions but dropped to 0.10 to 0.20 in damp ones. The composite’s SWR was measured at roughly 2–4 x 10^3^ mm^3^/Nm when dry but dropped dramatically when wet. Wear velocity increases the number of micro‐cracks discovered at the specimen and the size of the wear debris. Microplowing occurred because of third‐body abrasion. The SWR of CFRP decreased with increasing velocity in the wet state. This was because there was a quicker decrease in abrasiveness with increasing speed. An interesting conclusion that can be drawn from the above two studies is that the COF versus SD under dry conditions was comparatively lower for the betel nut fiber composite than that of the CFRP composite. The reason might be attributed to the inherent presence of wax and natural oils in betel nut fibers that lowers the COF in betel nut composites.

**FIGURE 5:**
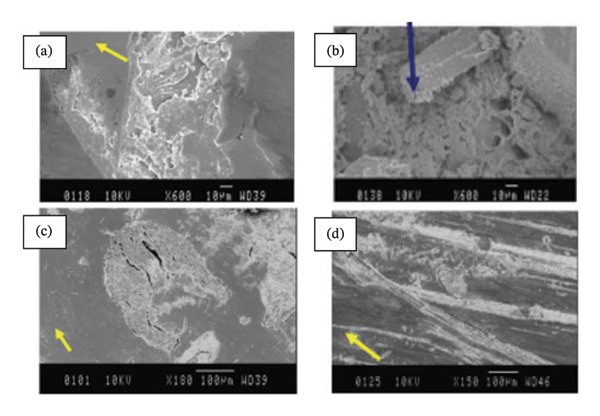
SEM micrographs of the treated and untreated OPRP composite tested using POD at 2.8 m/s sliding velocity for different sliding distances—5 km for (a) & (b) and 10 km for (c) & (d), respectively.

In 2009, Yousif and El‐Tayeb [73] conducted a study on the wear behavior of two types of oil palm fiber–reinforced polymer (OPRP) composites: untreated OPRP composite and treated OPRP composite. The investigation employed two distinct techniques to assess tribological behavior under wet contact conditions: the block on ring (BOR) method and the pin on disc (POD) method. Results indicated that the SWR value for the treated OPRP composite was significantly lower than that of the untreated OPRP composite. Furthermore, the introduction of water at the contact surfaces resulted in a notable reduction in wear when compared to the findings of the prior study. The researchers discovered that the soil water repellency of the treated specimens fell between the respective ranges of 70% and 43.5% when assessed using the BOR and POD test techniques. This phenomenon can be attributed to the fact that the sample with a variable interaction area exhibits a higher material removal rate. The wear occurring in both the treated and untreated composites shows that for a 5 km SD, the wear occurring in the treated composite is lower compared to the nontreated composite. A similar trend follows for a 10 km SD. It is interesting to note that higher wear is noted for nontreated composites at a 5 km SD compared to treated composites even at a 10 km SD. This is attributed to good fiber matrix bonding in treated composites, resulting in lower wear in composites. Figure 5 depicts the wear behavior in the developed composite.

Under wet contact conditions, Yousif et al. [74] investigated the frictional behavior and adhesive wear of polyester composites manufactured from betel nut fiber mats. The adhesive wear and frictional behavior of two additional fiber mats and the betel nut fiber reinforcement composite were examined. When tested in a POD machine against a stainless steel counterface at varying SVs (2.8 m/s), distances (0–6.72 km), and ALs (20–200 N), betel nut fiber reinforcement polymer composites in both P‐O and N‐O exhibit the same trend. A SD of 2.52 km is needed to reach a steady state, and an applied force of 30 N produced the maximum SWR. Similarly large loads of 70 N, 130 N, and 200 N were also necessary. The results of the study, which looked at both wet and dry conditions, indicate that parallel orientation has values for specific wear of 85%–90%, while normal orientation has the greatest values at 93%–100%. It was discovered that the friction coefficient varied more than usual for normal orientation while sliding, suggesting that the polyester or fiber may have come into contact with the counterface. Interestingly, the friction coefficient decreased to 91% during wet sliding. The majority of the wear was a result of the fibers being loose and getting trampled. At greater strains, micro and macro cracking in the resinous area was found. Dugarjav et al. [75] conducted a short communication on frictional performance on rice husk (RH)–reinforced phenolic resin composites under dry and lubricated conditions. Using a BOD apparatus, the friction and wear parameters of RH composites were tested in dry and water‐lubricated conditions against austenitic stainless steel (JIS SUS304). Dry conditions were found to result in lower friction coefficients and SWRs for RH composites than wet conditions. Dry conditions resulted in a friction coefficient of 0.06–0.11, whereas wet conditions resulted in a coefficient of 0.09–0.15. In both cases, the particular wear rate was less than 108 mm2/N, with the dry condition being significantly better. Micrographs of the ball’s damaged surface in dry conditions revealed the presence of transferred components. Conversely, in a water‐lubricated state, fewer transferred materials were visible on the worn surface. The microstructure of the transferred layer formed on opposite surfaces was shown to be associated with the observed difference in frictional behavior between dry and water‐lubricated conditions. The COF and SWR under dry sliding conditions were both significantly reduced due to the amorphous silica‐rich transferred film. As a result of the creation of a carbon‐dominated transferred coating in the presence of water, the COF and SWR of the RH composite were found to be greater than they had been under dry conditions. Nirmal et al. [76] carried out studies on the impact of mat‐form betel nut fibers in polyester composites. It was observed that SWR gradually decreased as SD grew and that this trend persisted until a fixed state transition. In comparison to its dry sliding state, the SWR reduced by around 10 times in the AP‐O and P‐O directions when the composite was wet, but it only decreased by about five times in the N‐O direction. Furthermore, in AP‐O, the wet condition resulted in lower SWR and COF values than the dry state. However, as observed from the SEM images as shown in Figure 6, the debonding is less in the case of treated composites compared to untreated ones in both dry/wet cases. This might be due to the improved interfacial adhesion between the fiber and matrix that resulted in lower fiber pullout. In the case of AP‐O, loose fibers that were visible on the composite’s worn exteriors are combined with fiber debonding and detachment. These fibers were found on the composite’s exterior. In the case of P‐O, the damage was brought on by both macro and microcracks, as well as the accompanying debonding and fiber separation.

FIGURE 6SEM micrographs for single fiber pullout test under dry/wet condition: (a) fiber pullout after dry test and (b) fiber pullout after wet test.

**Figure:**
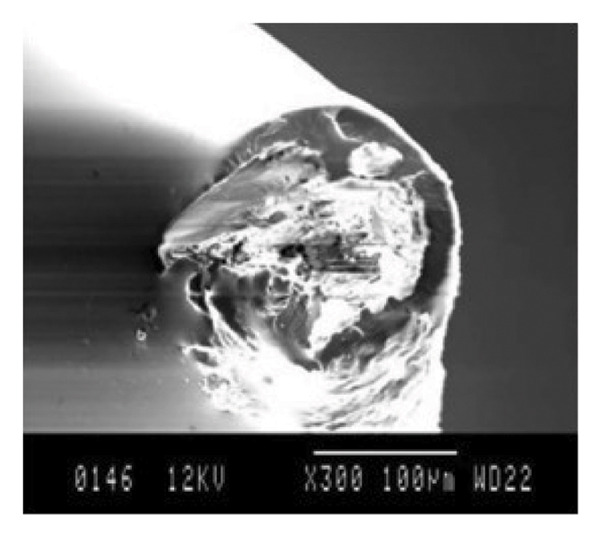
(a)

**Figure:**
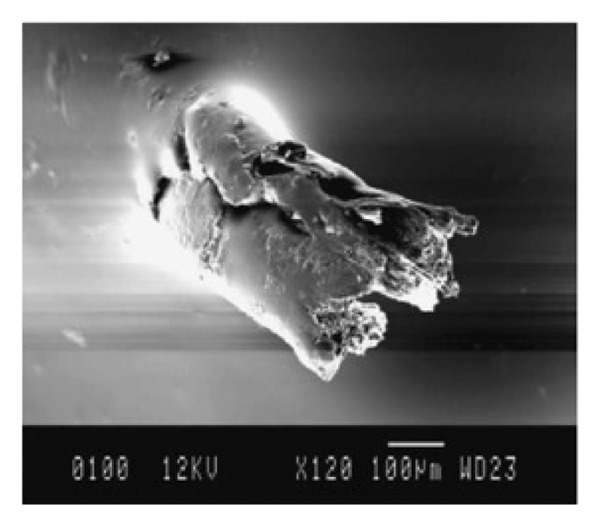
(b)

Yousif and Nirmal [77] examined the tribological performance of palm fiber polymeric composites aged in various solutions. For three years, the composite samples were immersed in a variety of liquids, such as fresh water, salt water, diesel, gasoline, and motor oil. Using a POD machine, wear experiments on treated oil palm fiber–reinforced composites submerged in both fresh and saline water revealed that, at 2.8 m/s, the specific wear rose from 2.5 to 4 km. When treated oil palm fiber–reinforced composites were submerged in water, their SWR decreased by around 18%, but at 5 km, a stable condition of SWR was reached when the composites were submerged in motor oil, diesel, or gasoline. This could be as a result of the cell wall densification that occurred in the fibers exposed to 50% salt water, which made them brittle in the resin. Compared to diesel or motor oil, the oil palm fiber–reinforced composite’s SWR increased when treated with gasoline. The thermomechanical strain on this composite is lessened when there is engine oil at the interface during sliding. Among the five submerged solutions, diesel exhibited the highest frictional performance, trailed by motor oil, water, petrol, and salt water. Narish et al. [78] researched to investigate the tribological behavior of kenaf fiber in wet contact scenarios. The standing wave ratio (SWR) falls with increasing contact area in damp contact circumstances. It was demonstrated in the AP‐O and N‐O studies that samples under high load had the lowest rate of wear. The results showed that when the load rose, the average COF decreased. When the composite material is exposed to wet conditions instead of dry ones, its frictional performance significantly improves by more than 90%. Water’s ability to lessen the COF and surface temperature of the sliding surface is the reason for this phenomenon. The worn surfaces of the wet contact specimen showed relatively lower levels of damage, according to the microscopic analysis that was done. In addition, there were no instances of fiber pull‐out or microcracks, and the degree of plastic deformation was determined to be less noticeable. Water was essential in preserving the interface’s temperature and preventing composite deformation by postponing thermal deterioration. Yousif and Chin [79] carried out a study focusing on changing fiber orientation that examined the adhesive wear behavior and frictional characteristics of Kenaf fiber–reinforced epoxy (KFRE) under wet conditions. According to the experimental results, the friction coefficient values ranged from 0.03 to 0.045, with the AP‐O condition exhibiting the lowest value. The liquid inside the boundary is responsible for this phenomenon because it helps to remove entangled bits from the shearing zone. The COF is thought to be decreasing because of this event. In comparison to the wear resistance of the AP and P‐O materials, the wear resistance of the KFRE composite showed a significant increase of around 35%–57%. Within the framework of P‐O, the fiber peeling phenomenon can be explained by the existence of a parallel‐acting shear force. The study revealed that the fiber located in the N‐O region experiences comparatively lower damage compared to the PO region, as well as in the AP‐O region. Additionally, no instances of debonding, pull‐out, or peeling were observed in N‐O. Paul and Bhowmik [80] examined the epoxy composite reinforced with coir filler’s wear performance in hot, humid, and dry environments. In the studies, three factors were tested: normal load (5–40 N), SV (100 cm/s), and SD (0–2000 m). The results showed that the wear behavior of the coir filler–reinforced composite was significantly influenced by the operating parameters and the filler reinforcement. In damp conditions, the SWR is lower than in dry and warm contact. Integrating coir filler into the polymer material shows a solid link between the matrix and filler, which results in a coir filler–reinforced polymer composite with superior adhesive wear representation than the virgin polymer sample. High volume loss is seen in pristine epoxy material under the dry sliding condition. The SWR improves during the break‐in period and reaches a constant level eventually afterward. Comparing simple epoxy in dry contact with all normal loads, the volume loss is significantly lessened. Consequently, in a wet contact scenario, the treated composite’s SWR is around seven to eight times lower than it is in a dry contact scenario. Volume loss increases with temperature in a hot contact state; however, above a particular temperature, the transfer of a thin film layer protects the composite’s surface from additional wear. SEM analysis reveals a large number of wear chips, microcracks, and clusters of filler material. In its dry form, epoxy reveals signs of significant material loss, including many surface fissures. In moist contact situations, wear debris production is greatly reduced due to the presence of water. When the contact is heated, a thin film of friction and micro‐plowing may be seen forming. The work was further extended by the authors [81] on the wear performance of bamboo filler–reinforced epoxy composite under dry, wet, and heated conditions. A study found that volume loss in wet conditions is remarkably low compared to dry and heated conditions. The composite with a bamboo filler loading of between 2.5 and 5 wt% has the lowest SWR value because the water used to make it also serves to generate a lubricant coating on the surface, protecting it from wear and tear. The composite’s wear resistance was enhanced due to the presence of water, which decreased the thermo‐mechanical loading. The SWR is also improved when using support with 10 or 12.5 wt% bamboo particles. The COF decreases by 1.2–3.0 times for plain epoxy and by 1.5–3.0 times for a composite with different amounts of bamboo filler when wet, compared to when both are dry and subjected to a force of 20 N. That peripheral lubricant introduction is sanctioned. The microscopic scans show that the fillers have not broken down and that there has been minimal distortion of the resin. Because of the steady flow of water, the outside never got hot enough to reduce COF, and vice versa. When the SD is increased, a thicker boundary layer forms on the surface, protecting the composite surface and reducing friction. The work was further extended by the above authors [82] on wood filler–reinforced epoxy composite under different contact conditions. The SWR and COF for the material are lower when the filler stuffing is lower (2.5, 5, and 7.5 wt%) than when the filler reinforcement is higher (10 and 12.5 wt%). The amount of material lost in the wet condition is significantly less since coolant is included throughout the testing procedure. In this instance, compared to the dry condition, the wood dust–reinforced composite’s SWR is cut by 80%–90%, and its COF is lowered by 50%–60%. If the temperature rises to a high enough level, a back‐film transfer will occur throughout the composite surface. This prevents additional surface deterioration, and when the system is heated, the SWR and COF drop by 35%–40% and 15%–20%, respectively, in comparison to the dry state. The removal of bulk material has left the clean epoxy with numerous fissures. Plastic deformation is essentially nonexistent when the material is wet due to the incorporation of the cooling media.

### 4.2. Dry Adhesive Test

A dry sliding wear test is a method used to assess the frictional and wear properties of materials when they slide against each other in the absence of any lubricant or coolant. This type of test is particularly relevant in industries where components are subjected to sliding motion under dry conditions, such as automotive, aerospace, and manufacturing.

A work reported by Sharma et al. [82] reveals the tribological behavior of food waste (*Citrus limetta* peel) filler–reinforced epoxy composites under dry conditions. The composite was developed using 15% by weight of filler in epoxy resin with three different particle size ranges of 100–250 μm, 350–500 μm, and 650–800 μm. The study reveals that the incorporation of CLP fillers in epoxy matrix improves the wear resistance of the developed composites. Further, the highest wear resistance was observed for fillers with a 100–250 μm size. A study by Murali et al. [83] shows the dry sliding wear behaviors of hybrid polymer matrix composites with epoxy as the matrix material and Kevlar, bamboo, palm, and *Aloe vera* as reinforcement materials in different stacking sequences. D‐optimal design and analysis of variance based on the response surface approach were used to design experiments and carry out statistical validation. The ideal parameters were determined to be a load of 5 N, a SV of 3 m/s, and a SD of 1500 m for composite combinations of CA among AB, BC, and CA. Additional ANOVA results showed that the suggested quadratic mathematical models are effective in predicting the wear behaviors of manufactured composites at 95% conformance levels, with a coefficient of determination of 97.09% for COF and 98.92% for SWR. A work conducted by Yadav et al. [84] reported that the wear properties of fabricated sisal, banana, and bagasse fiber–reinforced PLA composites were investigated on a digital display–controlled pin‐on‐disc test rig under dry sliding conditions. The SD was 1000–3000 m, the sliding speed was 2.4 and 6 m/s, and the AL was 10 N. The authors observed that there was a decrease in wear rate and frictional coefficient with an increase in fiber concentration from 10% to 20%. Further chemical treatment of fillers helped in lowering the wear rate and friction coefficient of composites.

### 4.3. Critical Role of Filler Particle Size and Morphology in Three‐Body Abrasive Wear of Natural Fiber–Reinforced Polymer (NFRP) Composites

A significant finding in the literature, particularly under three‐body abrasive wear conditions, is the critical influence of filler particle size and morphology within the polymer matrix. When fine, hard debris particles (the third body) are introduced between the rubbing surfaces, their ability to embed into the composite matrix—and consequently cause material removal—is heavily dictated by the characteristics of the reinforcing material. Research has shown that composites incorporating micro‐sized fillers often exhibit greater resistance to three‐body abrasion compared to those with nanosized or coarse fillers [85, 86]. This is primarily because micro‐sized particles can effectively harden the surface layer without introducing excessive porosity, which can act as nucleation sites for crack initiation or allow the easy accommodation of abrasive particles. Furthermore, the aspect ratio and surface roughness of the natural fiber fillers significantly modulate the wear mechanism. Fibers with higher aspect ratios, when oriented appropriately, can share the AL more efficiently, shielding the matrix from direct contact and particle indentation. Conversely, fillers with irregular or highly rough morphologies can themselves become stress concentrators, leading to preferential wear paths under abrasive loading. Therefore, optimizing the geometrical properties of the natural filler is as crucial as optimizing its volume fraction when designing NFRP composites for applications where three‐body abrasion is a predominant wear mode [87, 88].

### 4.4. Inferences Drawn From the Aforesaid Tests

In contrast, there is not much work that involves testing in a wet contact environment compared to the dry sliding test. It has been shown that any lubricating medium during sliding minimizes wear and friction by acting as a coolant during the sliding process. It prevents the rubbing interface from becoming too hot, which lessens the composite’s exposure to thermomechanical loading. Furthermore, coolant washes away wear debris, which helps maintain a pristine rubbing interface. Wet sliding tests show more wear and friction improvements than dry sliding tests. The constant inclusion of lubricant between the sample and counterface surface during wet contact kept the interface temperature low and produced a very low SWR. Due to the constant supply of coolant medium, the COF is also very low in wet contact conditions. However, an increase in wear rate and COF occurs due to the non‐availability of coolant in the dry test. This further reduces its usage in measuring tribological properties of composites.

## 5. Conclusion

This review provided a comprehensive examination of the tribological performance of NFRP composites, with a focus on adhesive and abrasive wear under lubricated (wet), dry, and heated contact conditions. The literature consistently demonstrates that NFRP composites, which are a sustainable and cost‐effective alternative to synthetic materials, are highly sensitive to both material and operational parameters in tribological applications. Key findings from the review include the following [89].

• The presence of a liquid medium (wet contact) consistently and significantly improves the tribological performance of NFRP composites, resulting in a drastically lower SWR and COF compared to dry or heated conditions.
• The liquid serves multiple beneficial roles: It acts as a coolant to reduce interface temperature, minimizes thermo‐mechanical loading on the composite, and effectively flushes away wear debris, thereby maintaining a clean rubbing interface and preventing severe damage like fiber pull‐out or microcracks.
• Chemical treatments are crucial for enhancing the composite’s wear resistance. Treated NFRP composites exhibit superior performance (lower SWR) compared to untreated ones, which is attributed to an improved interfacial adhesion (fiber–matrix bonding).
• Fiber orientation is a critical factor, with AP‐O and N‐O often providing better wear resistance and lower friction than P‐O, which is more prone to fiber peeling and greater surface damage.
• Tribological properties are significantly affected by material parameters (e.g., particle size, volume fraction, fiber length) and operating parameters (e.g., AL, SV, SD, and temperature).

## Author Contributions

Rajdeep Paul, Sumit Das Lala, and Payel Deb: writing–original draft; Abhijit Bhowmik and Nagaraj Ashok: formal analysis and proofreading.

## Funding

The study did not receive any funding.

## Conflicts of Interest

The authors declare no conflicts of interest.

## Data Availability

The data that support the findings of this study are available from the corresponding author upon reasonable request.

## References

[1] Mohanty A. K. , Misra M. , and Drzal L. T. , Sustainable Biocomposites from Renewable Resources: Opportunities and Challenges in the Green Materials World, Journal of Polymers and the Environment. (2002) 10, no. 2, 19–26, 10.1023/a:1021013921916, 2-s2.0-0347472168.

[2] Gopan G. , Arun M. , Adel M. , Khader M. M. , and Ahmad H. , Investigation of Multi-Modal Binder Optimization Characterization Modeling by Using Metal Oxide Nanoparticles, BioNanoScience. (2025) 15, no. 1, 10.1007/s12668-024-01750-0.

[3] Drzal L. T. , Mohanty A. K. , and Misra M. , Biocomposite Materials as Alternatives to Petroleum-based Composites for Automotive Applications, Magnesium. (2001) 40, 1–3.

[4] Koronis G. , Silva A. , and Fontul M. , Green Composites: A Review of Adequate Materials for Automotive Applications, Composites, Part B: Engineering. (2013) 44, no. 1, 120–127, 10.1016/j.compositesb.2012.07.004, 2-s2.0-84867464787.

[5] Bhowmik A. , Nithin A. M. , Kumar R. et al., Prediction Friction Forecasting in AA7075 Composites Through Ensemble and Probabilistic Machine Learning, Journal of Sol-Gel Science and Technology. (2025) 116, no. 3, 1–17, 10.1007/s10971-025-07000-3.

[6] Larsen T. , Andersen T. L. , Thorning B. , Horsewell A. , and Vigild M. E. , Comparison of Friction and Wear for an Epoxy Resin Reinforced by a Glass or a carbon/aramid Hybrid Weave, Wear. (2007) 262, no. 8, 1013–1020, 10.1016/j.wear.2006.10.004, 2-s2.0-33847083554.

[7] Bhowmik A. , Rachchh N. , Patil N. et al., Synthesis and Evaluation of Aluminium Matrix Composites Reinforced with Laboratory Waste Borosilicate Glass, Discover Sustainability. (2025) 6, no. 1, 10.1007/s43621-025-01937-9. PMC13493849 42218243

[8] Wan Y. Z. , Huang Y. , He F. , Li Q. , and Lian J. , Tribological Properties of three-dimensional Braided Carbon/Kevlar/Epoxy Hybrid Composites Under Dry and Lubricated Conditions, Materials Science and Engineering: A. (2007) 453, 202–209, 10.1016/j.msea.2006.11.090, 2-s2.0-33847250398.

[9] Paul R. , Gouda K. , and Bhowmik S. , Effect of Different Constraint on Tribological Behaviour of Natural Fibre/Filler Reinforced Polymeric Composites: A Review, Silicon. (2021) 13, no. 8, 2785–2807, 10.1007/s12633-020-00613-z.

[10] Teli G. , Mahakur V. K. , Paul R. , and Bhowmik S. , Investigation of Dry Sliding Tribological Behaviour of Epoxy Composites Filled with Hemp Particulates Using Artificial Neural Networks, Arabian Journal for Science and Engineering. (2023) 48, no. 3, 3989–4001, 10.1007/s13369-022-07354-8.

[11] Olusegun D. S. , Stephen A. , and Adekanye T. A. , Assessing Mechanical Properties of Natural Fibre Reinforced Composites for Engineering Applications, Journal of Minerals and Materials Characterization and Engineering. (2012) 11, 780–784.

[12] Ray S. S. and Bousmina M. , Biodegradable Polymers and Their Layered Silicate Nanocomposites: In Greening the 21st Century Materials World, Progress in Materials Science. (2005) 50, 962–1079.

[13] Al-Oqla F. M. and Sapuan S. M. , Natural Fiber Reinforced Polymer Composites in Industrial Applications: Feasibility of Date Palm Fibers for Sustainable Automotive Industry, Journal of Cleaner Production. (2014) 66, 347–354, 10.1016/j.jclepro.2013.10.050, 2-s2.0-84893791582.

[14] Hänninen T. , Thygesen A. , Mehmood S. , Madsen B. , and Hughes M. , Mechanical Processing of Bast Fibres: The Occurrence of Damage and its Effect on Fibre Structure, Industrial Crops and Products. (2012) 39, 7–11, 10.1016/j.indcrop.2012.01.025, 2-s2.0-84857267420.

[15] Van de Weyenberg I. , Ivens J. , De Coster A. , Kino B. , Baetens E. , and Verpoest I. , Influence of Processing and Chemical Treatment of Flax Fibres on Their Composites, Composites Science and Technology. (2003) 63, no. 9, 1241–1246, 10.1016/s0266-3538(03)00093-9, 2-s2.0-0038205468.

[16] Thakur V. K. and Thakur M. K. , Processing and Characterization of Natural Cellulose Fibers/thermoset Polymer Composites, Carbohydrate Polymers. (2014) 109, 102–117, 10.1016/j.carbpol.2014.03.039, 2-s2.0-84898620127. 24815407

[17] Dai D. and Fan M. , Wood Fibres as Reinforcements in Natural Fibre Composites: Structure, Properties, Processing and Applications, Natural Fibre Composites. (2014) Woodhead Publishing, 3–65.

[18] Sreekala M. S. , Kumaran M. G. , and Thomas S. , Stress Relaxation Behaviour in Oil Palm Fibres, Materials Letters. (2001) 50, no. 4, 263–273, 10.1016/s0167-577x(01)00237-3, 2-s2.0-0035446968.

[19] Jayamani E. , Hamdan S. , Rahman M. R. , and Bakri M. K. B. , Investigation of Fiber Surface Treatment on Mechanical, Acoustical and Thermal Properties of Betelnut Fiber Polyester Composites, Procedia Engineering. (2014) 97, 545–554, 10.1016/j.proeng.2014.12.282, 2-s2.0-84922337050.

[20] Pan Y. and Zhong Z. , A Micromechanical Model for the Mechanical Degradation of Natural Fiber Reinforced Composites Induced by Moisture Absorption, Mechanics of Materials. (2015) 85, 7–15, 10.1016/j.mechmat.2015.02.001, 2-s2.0-84939214932.

[21] Ren B. , Mizue T. , Goda K. , and Noda J. , Effects of Fluctuation of Fibre Orientation on Tensile Properties of Flax Sliver-Reinforced Green Composites, Composite Structures. (2012) 94, no. 12, 3457–3464, 10.1016/j.compstruct.2012.06.002, 2-s2.0-84864770028.

[22] Ramesh M. , Atreya T. S. A. , Aswin U. S. , Eashwar H. , and Deepa C. , Processing and Mechanical Property Evaluation of Banana Fiber Reinforced Polymer Composites, Procedia Engineering. (2014) 97, 563–572, 10.1016/j.proeng.2014.12.284, 2-s2.0-84922334137.

[23] Boopathi L. , Sampath P. S. , and Mylsamy K. , Investigation of Physical, Chemical and Mechanical Properties of Raw and Alkali Treated Borassus Fruit Fiber, Composites, Part B: Engineering. (2012) 43, no. 8, 3044–3052, 10.1016/j.compositesb.2012.05.002, 2-s2.0-84866736841.

[24] Manfredi L. B. , Rodríguez E. S. , Wladyka-Przybylak M. , and Vázquez A. , Thermal Degradation and Fire Resistance of Unsaturated Polyester, Modified Acrylic Resins and Their Composites with Natural Fibres, Polymer Degradation and Stability. (2006) 91, no. 2, 255–261, 10.1016/j.polymdegradstab.2005.05.003, 2-s2.0-27844549440.

[25] Célino A. , Fréour S. , Jacquemin F. , and Casari P. , The Hygroscopic Behavior of Plant Fibers: A Review, Frontiers of Chemistry. (2014) 1, 10.3389/fchem.2013.00043, 2-s2.0-84986625658. PMC3982556 24790971

[26] Methacanon P. , Weerawatsophon U. , Sumransin N. , Prahsarn C. , and Bergado D. T. , Properties and Potential Application of the Selected Natural Fibers as Limited Life Geotextiles, Carbohydrate Polymers. (2010) 82, no. 4, 1090–1096, 10.1016/j.carbpol.2010.06.036, 2-s2.0-77956493521.

[27] Wongsriraksa P. , Togashi K. , Nakai A. , and Hamada H. , Continuous Natural Fiber Reinforced Thermoplastic Composites by Fiber Surface Modification, Advances in Mechanical Engineering. (2013) 5, 10.1155/2013/685104, 2-s2.0-84876533914.

[28] Gassan J. and Bledzki A. K. , Possibilities for Improving the Mechanical Properties of Jute/Epoxy Composites by Alkali Treatment of Fibres, Composites Science and Technology. (1999) 59, no. 9, 1303–1309, 10.1016/s0266-3538(98)00169-9, 2-s2.0-0032860002.

[29] Rong M. Z. , Zhang M. Q. , Liu Y. , Yang G. C. , and Zeng H. M. , The Effect of Fiber Treatment on the Mechanical Properties of Unidirectional Sisal-Reinforced Epoxy Composites, Composites Science and Technology. (2001) 61, no. 10, 1437–1447, 10.1016/s0266-3538(01)00046-x, 2-s2.0-0035423018.

[30] Hossain S. I. , Hasan M. , Hasan M. N. , and Hassan A. , Effect of Chemical Treatment on Physical, Mechanical and Thermal Properties of Ladies Finger Natural Fiber, Advances in Materials Science and Engineering. (2013) 2013, 1–6, 10.1155/2013/824274, 2-s2.0-84886693270.

[31] Tran T. P. T. , Bénézet J. C. , and Bergeret A. , Rice and Einkorn Wheat Husks Reinforced Poly (Lactic Acid) (PLA) Biocomposites: Effects of Alkaline and Silane Surface Treatments of Husks, Industrial Crops and Products. (2014) 58, 111–124, 10.1016/j.indcrop.2014.04.012, 2-s2.0-84899799177.

[32] Luo H. , Xiong G. , Ma C. et al., Mechanical and thermo-mechanical Behaviors of sizing-treated Corn fiber/polylactide Composites, Polymer Testing. (2014) 39, 45–52, 10.1016/j.polymertesting.2014.07.014, 2-s2.0-84905827839.

[33] O′donnell A. , Dweib M. A. , and Wool R. P. , Natural Fiber Composites With Plant Oil-Based Resin, Composites Science and Technology. (2004) 64, 1135–1145.

[34] Paul A. , Joseph K. , and Thomas S. , Effect of Surface Treatments on the Electrical Properties of Low-Density Polyethylene Composites Reinforced With Short Sisal Fibers, Composites Science and Technology. (1997) 57, no. 1, 67–79, 10.1016/s0266-3538(96)00109-1, 2-s2.0-0030924670.

[35] Ismail H. , Rusli A. , and Rashid A. A. , Maleated Natural Rubber as a Coupling Agent for Paper Sludge Filled Natural Rubber Composites, Polymer Testing. (2005) 24, no. 7, 856–862, 10.1016/j.polymertesting.2005.06.011, 2-s2.0-25644442477.

[36] Torres F. G. and Cubillas M. L. , Study of the Interfacial Properties of Natural Fibre Reinforced Polyethylene, Polymer Testing. (2005) 24, no. 6, 694–698, 10.1016/j.polymertesting.2005.05.004, 2-s2.0-23844547436.

[37] Corrales F. , Vilaseca F. , Llop M. , Girones J. , Mendez J. A. , and Mutje P. , Chemical Modification of Jute Fibers for the Production of Green-Composites, Journal of Hazardous Materials. (2007) 144, no. 3, 730–735, 10.1016/j.jhazmat.2007.01.103, 2-s2.0-34249060960. 17320283

[38] He L. , Li X. , Li W. , Yuan J. , and Zhou H. , A Method for Determining Reactive Hydroxyl Groups in Natural Fibers: Application to Ramie Fiber and its Modification, Carbohydrate Research. (2012) 348, 95–98, 10.1016/j.carres.2011.10.035, 2-s2.0-84856233302. 22099251

[39] Hidayat A. and Tachibana S. , Characterization of Polylactic Acid (PLA)/Kenaf Composite Degradation by Immobilized Mycelia of Pleurotus ostreatus, International Biodeterioration & Biodegradation. (2012) 71, 50–54, 10.1016/j.ibiod.2012.02.007, 2-s2.0-84860876518.

[40] Xie K. , Liu H. , and Wang X. , Surface Modification of Cellulose With Triazine Derivative to Improve Printability With Reactive Dyes, Carbohydrate Polymers. (2009) 78, no. 3, 538–542, 10.1016/j.carbpol.2009.05.013, 2-s2.0-68949099595.

[41] Kabir M. M. , Wang H. , Lau K. T. , and Cardona F. , Chemical Treatments on Plant-based Natural Fibre Reinforced Polymer Composites: An Overview, Composites, Part B: Engineering. (2012) 43, no. 7, 2883–2892, 10.1016/j.compositesb.2012.04.053, 2-s2.0-84864288656.

[42] Mohammed L. , Ansari M. N. , Pua G. , Jawaid M. , and Islam M. S. , A Review on Natural Fiber Reinforced Polymer Composite and its Applications, International Journal of Polymer Science. (2015) 2015, 1–15, 10.1155/2015/243947, 2-s2.0-84944229020.

[43] Paul R. and Bhowmik S. , Effect of Load on Wear Performance of Coir Particulate Reinforced Epoxy Composite, AIP Conference Proceedings, 2019, 2200, no. 1, AIP Publishing, 10.1063/1.5141269.

[44] Paul R. , Gouda K. , and Bhowmik S. , The Effect of Filler Treatment on the Frictional Performance of Coir Dust Reinforced Polymeric Composite, Materials Today: Proceedings. (2021) 46, 9079–9083, 10.1016/j.matpr.2021.05.391.

[45] Torabinejad V. , Aliofkhazraei M. , Assareh S. , Allahyarzadeh M. H. , and Sabour A. R. , Electrodeposition of Ni-Fe Alloys, Composites, and Nano coatings—A Review, Journal of Alloys and Compounds. (2017) 691, 841–859, 10.1016/j.jallcom.2016.08.329, 2-s2.0-84986917887.

[46] Holmberg K. and Erdemir A. , Influence of Tribology on Global Energy Consumption, Costs and Emissions, Friction. (2017) 5, no. 3, 263–284, 10.1007/s40544-017-0183-5, 2-s2.0-85029144747.

[47] Holmberg K. , Kivikytö-Reponen P. , Härkisaari P. , Valtonen K. , and Erdemir A. , Global Energy Consumption due to Friction and Wear in the Mining Industry, Tribology International. (2017) 115, 116–139, 10.1016/j.triboint.2017.05.010, 2-s2.0-85019720563.

[48] Bhushan B. , Introduction to Tribology, 2013, John Wiley & Sons Ltd, Hoboken, NJ, USA, 1–201.

[49] Milosevic M. , Valášek P. , and Ruggiero A. , Tribology of Natural Fibers Composite Materials: An Overview, Lubricants. (2020) 8, no. 4, 10.3390/lubricants8040042.

[50] Popov V. L. , Popov V. L. , Qualitative Treatment of Contact Problems—Normal Contact Without Adhesion, Contact Mechanics and Friction, Physical Principles and Applications, 2017, Springer, Berlin, Germany, 9–23.

[51] Popov V. L. , Heβ M. , and Willert E. , Handbook of Contact Mechanics, Exact Solutions of Axisymmetric Contact Problems, 2019, Springer, Berlin, Germany, 5–66.

[52] D’Agostino V. , Fondamenti Di Meccanica Applicata Alle Macchine, 2013, Maggioli Editore, Rimini, Italy, 124–138.

[53] Rahnejat H. , Rahnejat H. , An Introduction to multi-physics multi-scale Approach, Tribology and Dynamics of Engine and Powertrain, Fundamentals, Applications and Future Trends, 2010, Woodhead Publishing, Sawston, UK, 3–38.

[54] Rahnejat H. and Johns-Rahnejat P. M. , Mechanics of Contacting Surfaces, Encyclopedia of Automotive Engineering, 2014, John Wiley & Sons Ltd, Hoboken, NJ, USA, 1–9.

[55] Mahakur V. K. , Paul R. , Bhowmik S. , and Patowari P. K. , Influence of Surface Modification on Mechanical and Tribology Performance of Jute Filler Polymer Composites and Prediction of the Performance Using Artificial Neural Network, Polymer Bulletin. (2022) 80, no. 11, 1–22, 10.1007/s00289-022-04636-x.

[56] Boucly V. , Nélias D. , Liu S. , Wang Q. J. , and Keer L. M. , Contact Analyses for Bodies with Frictional Heating and Plastic Behavior, Journal of Tribology. (2005) 127, no. 2, 355–364, 10.1115/1.1843851, 2-s2.0-18644373018.

[57] Yevtushenko A. A. and Kuciej M. , One-Dimensional Thermal Problem of Friction During Braking: The History of Development and Actual State, International Journal of Heat and Mass Transfer. (2012) 55, no. 16, 4148–4153, 10.1016/j.ijheatmasstransfer.2012.03.056, 2-s2.0-84861527236.

[58] Liu C. R. and Guo Y. B. , Finite Element Analysis of the Effect of Sequential Cuts and tool–chip Friction on Residual Stresses in a Machined Layer, International Journal of Mechanical Sciences. (2000) 42, no. 6, 1069–1086, 10.1016/s0020-7403(99)00042-9, 2-s2.0-0033894531.

[59] Bassani R. , Levita G. , Meozzi M. , and Palla G. , Friction and Wear of Epoxy Resin on Inox Steel: Remarks on the Influence of Velocity, Load and Induced Thermal State, Wear. (2001) 247, no. 2, 125–132, 10.1016/s0043-1648(00)00498-1, 2-s2.0-0035241952.

[60] Páczelt I. and Mróz Z. , Variational Approach to the Analysis of steady-state thermo-elastic Wear Regimes, International Journal for Numerical Methods in Engineering. (2009) 81, no. 6, 728–760, 10.1002/nme.2709, 2-s2.0-74949137892.

[61] Lim S. C. and Ashby M. F. , Overview No. 55 Wear-Mechanism Maps, Acta Metallurgica. (1987) 35, 1–24, 10.1016/0001-6160(87)90209-4, 2-s2.0-0002049167.

[62] Amiri M. and Khonsari M. M. , On the Thermodynamics of Friction and wear—A Review, Entropy. (2010) 12, no. 5, 1021–1049, 10.3390/e12051021, 2-s2.0-77953518535.

[63] Paul R. , Zindani D. , and Bhowmik S. , Investigation on Physicomechanical, Tribological and Optimality Condition for Coir Filler-Reinforced Polymeric Composites, Arabian Journal for Science and Engineering. (2023) 48, no. 3, 3615–3630, 10.1007/s13369-022-07221-6.

[64] Suh N. P. , Tribophysics. Pretice-hall, 1986.

[65] Stevenson A. N. J. and Hutchings I. M. , Development of the Dry sand/rubber Wheel Abrasion Test, Wear. (1996) 195, no. 1-2, 232–240, 10.1016/0043-1648(96)06965-7, 2-s2.0-0030199067.

[66] Blickensderfer R. and Laird G. , A pin-on-drum Abrasive Wear Test and Comparison with Other Pin Tests, Journal of Testing and Evaluation. (1988) 16, no. 6, 516–526, 10.1520/jte11270j.

[67] Yousif B. F. , Nirmal U. , and Wong K. J. , Three-Body Abrasion on Wear and Frictional Performance of Treated Betelnut Fibre Reinforced Epoxy (T-BFRE) Composite, Materials and Design. (2010) 31, no. 9, 4514–4521, 10.1016/j.matdes.2010.04.008, 2-s2.0-77953542525.

[68] Pıhtılı H. and Tosun N. , Effect of Load and Speed on the Wear Behaviour of Woven Glass Fabrics and Aramid fibre-reinforced Composites, Wear. (2002) 252, no. 11-12, 979–984, 10.1016/s0043-1648(02)00062-5, 2-s2.0-0036601412.

[69] Mergler Y. J. , Schaake R. P. , and Huis A. J. , Material Transfer of POM in Sliding Contact, Wear. (2004) 256, no. 3-4, 294–301, 10.1016/s0043-1648(03)00410-1, 2-s2.0-1442264835.

[70] Nirmal U. , Hashim J. , and Lau S. T. W. , Testing Methods in Tribology of Polymeric Composites, International Journal of Mechanical and Materials Engineering. (2011) 6, 367–373.

[71] Yousif B. F. , Lau S. T. , and McWilliam S. , Polyester Composite Based on Betelnut Fibre for Tribological Applications, Tribology International. (2010) 43, no. 2, 503–511, 10.1016/j.triboint.2009.08.006, 2-s2.0-74449086362.

[72] Danaelan D. and Yousif B. F. , Adhesive Wear Performance of CFRP Multilayered Polyester Composites Under dry/wet Contact Conditions, Surface Review and Letters. (2008) 15, no. 06, 919–925, 10.1142/s0218625x08012116, 2-s2.0-58449103238.

[73] Yousif B. F. and El-Tayeb N. S. M. , Wet Adhesive Wear Characteristics of Untreated Oil Palm fibre-reinforced Polyester and Treated Oil Palm fibre-reinforced Polyester Composites Using the pin-ondisc and block-on-ring Techniques, Proceedings-Institution of Mechanical Engineers, Part J: J Eng Tribol. (2010) 224, no. 2, 123–131, 10.1243/13506501jet655, 2-s2.0-77249083108.

[74] Yousif B. F. , Devadas A. , and Yusaf T. F. , Adhesive Wear and Frictional Behavior of Multilayered Polyester Composite Based on Betelnut Fiber Mats Under Wet Contact Conditions, Surface Review and Letters. (2009) 16, no. 03, 407–414, 10.1142/s0218625x09012792, 2-s2.0-68349144456.

[75] Dugarjav T. , Yamaguchi T. , Shibata K. , and Hokkirigawa K. , Friction and Wear Properties of Rice Husk Ceramics Under Dry and Water Lubricated Conditions, Tribology Online. (2009) 4, no. 4, 78–81, 10.2474/trol.4.78.

[76] Nirmal U. , Yousif B. F. , Rilling D. , and Brevern P. V. , Effect of Betelnut Fibres Treatment and Contact Conditions on Adhesive Wear and Frictional Performance of Polyester Composites, Wear. (2010) 268, no. 11-12, 1354–1370, 10.1016/j.wear.2010.02.004, 2-s2.0-77951123510.

[77] Yousif B. F. and Nirmal U. , Wear and Frictional Performance of Polymeric Composites Aged in Various Solutions, Wear. (2011) 272, no. 1, 97–104, 10.1016/j.wear.2011.07.006, 2-s2.0-80052993289.

[78] Narish S. , Yousif B. F. , and Rilling D. , Investigations on Wear and Frictional Properties of Kenaf Fibre Polyurethane Composites Under Dry and Wet Contact Conditions, International Journal of Precision Technology. (2011) 2, no. 4, 375–387.

[79] Yousif B. F. and Chin C. W. , Epoxy Composite Based on Kenaf Fibers for Tribological Applications Under Wet Contact Conditions, Surface Review and Letters. (2012) 19, no. 6, 10.1142/s0218625x12500503, 2-s2.0-84867174061.

[80] Paul R. and Bhowmik S. , Tribological Behavior of Micro Coir Filler Reinforced Polymer Composite Under Dry, Wet, and Heated Contact Condition, Journal of Natural Fibers. (2022) 19, no. 6, 2077–2092, 10.1080/15440478.2020.1798845.

[81] Paul R. and Bhowmik S. , Adhesive Wear Behaviour of Surface Modified Bamboo Filler Reinforced Polymer Composite Under Different Contact Condition, Journal of Natural Fibers. (2022) 19, no. 15, 12208–12223, 10.1080/15440478.2022.2054893.

[82] Paul R. and Bhowmik S. , Investigation of Mechanical and Tribological Performance of Wood Dust Reinforced Epoxy Composite Under Dry, Wet and Heated Contact Condition, International Polymer Processing. (2024) 39, no. 2, 186–201, 10.1515/ipp-2023-4410.

[83] Sharma H. , Misra J. P. , and Singh I. , Friction and Wear Behaviour of Epoxy Composites Reinforced with Food Waste Fillers, Composites Communications. (2020) 22, 10.1016/j.coco.2020.100436.

[84] Murali B. , Ramnath B. M. V. , Rajamani D. et al., Experimental Investigations on Dry Sliding Wear Behavior of Kevlar and Natural Fiber-Reinforced Hybrid Composites Through an RSM–GRA Hybrid Approach, Materials. (2022) 15, 10.3390/ma15030749. PMC8836847 35160692

[85] Srinivasan V. , Asaithambi B. , Ganesan G. , Karthikeyan R. , and Palanikumar K. , Wear Mechanism of Glass Fiber Reinforced Epoxy Composites Under Dry Sliding Using Fuzzy Clustering Technique, Journal of Reinforced Plastics and Composites. (2009) 28, no. 11, 1349–1358, 10.1177/0731684408089489, 2-s2.0-67649563662.

[86] Suresha B. , Vidyashree S. , and Bettegowda H. , Effect of Filler Materials on Abrasive Wear Performance of Glass/Epoxy Composites, Tribology in Industry. (2023) 44, no. 1, 111–120, 10.24874/ti.1386.10.22.01.

[87] Ramakrishnan S. , Krishnamurthy K. , Rajasekar R. , and Rajeshkumar G. , An Experimental Study on the Effect of nano-clay Addition on Mechanical and Water Absorption Behaviour of Jute Fibre Reinforced Epoxy Composites, Journal of Industrial Textiles. (2019) 49, no. 5, 597–620, 10.1177/1528083718792915, 2-s2.0-85052329410.

[88] Laranjeira E. , De Carvalho L. H. , De L Silva S. M. , and d’Almeida J. R. M. , Influence of Fiber Orientation on the Mechanical Properties of polyester/jute Composites, Journal of Reinforced Plastics and Composites. (2006) 25, no. 12, 1269–1278, 10.1177/0731684406060577, 2-s2.0-33747591435.

[89] Yadav V. , Singh S. , Chaudhary N. et al., Dry Sliding Wear Characteristics of Natural Fibre Reinforced poly-lactic Acid Composites for Engineering Applications: Fabrication, Properties and Characterizations, Journal of Materials Research and Technology. (2023) 23, 1189–1203, 10.1016/j.jmrt.2023.01.006.

